# Editorial: Toxicity mechanism and clinical features of PD-1/PD-l1 inhibitors in treatment of cancer, volume II

**DOI:** 10.3389/fphar.2023.1136373

**Published:** 2023-02-06

**Authors:** Chuqi Lei, Xiangyi Kong, Ryan J. Sullivan, Caisheng Wu, Dawei Wang, Lin Zhang

**Affiliations:** ^1^ Department of Breast Surgical Oncology, National Cancer Center, National Clinical Research Center for Cance, Cancer Hospital, Chinese Academy of Medical Sciences and Peking Union Medical College, Beijing, China; ^2^ Department of Breast Surgical Oncology, National Cancer Center, National Clinical Research Center for Cancer, Cancer Hospital and Shenzhen Hospital, Chinese Academy of Medical Sciences and Peking Union Medical College, Shenzhen, China; ^3^ Center for Melanoma, Massachusetts General Hospital Cancer Center, Harvard Medical School, Harvard University, Boston, MA, United States; ^4^ Laboratory Animal Center, Xiamen University, Xiamen, China; ^5^ School of Pharmaceutical Sciences, Xiamen University, Xiamen, China; ^6^ Department of Urology, Ruijin Hospital, School of Medicine, Shanghai Jiaotong University, Shanghai, China; ^7^ Institute of Artificial Intelligence, Hefei Comprehensive National Science Center, Hefei, China; ^8^ Suzhou Industrial Park Monash Research Institute of Science and Technology, Suzhou, China; ^9^ School of Population Medicine and Public Health, Peking Union Medical College, Chinese Academy of Medical Sciences, Beijing, China

**Keywords:** PD-1/PD-L1 inhibitors, toxicity mechanism, clinical features, immunotherapy, cancer

The Research Topic titled “*Toxicity Mechanism and Clinical features of PD-1/PD-L1 Inhibitors in Treatment of Cancer, Volume II*” is part of a series with “Toxicity Mechanism and Clinical features of PD-1/PD-L1 Inhibitors in Treatment of Cancer, Volume I”. Building on the previous section, this Research Topic focused on exploring the mechanism of pharmacological toxicity induced by PD-1/PD-L1 inhibitors and the difference in the incidence of toxicities between patients treated with PD-1 or PD-L1 inhibitors and those treated without. PD-1 and PD-L1 inhibitors are important targets in immunotherapy for cancer treatment. Although the promising efficacy of Anti-PD-1/PD-L1 antibodies has been confirmed, their concomitant toxic effects are a negative factor affecting the efficacy. It is necessary to conduct in-depth research on the mechanisms and clinical manifestations of immune adverse reactions. The eight articles published in this section are the remarkable masterpiece provided by dozens of teams of experts from many countries and regions, including four reviews, one case report, and three original studies. Covering a wide range of solid tumors, it presents the latest insights from various frontline expert teams.

From the very beginning, immune-related adverse effects (irAEs) are known to be organ-specific and can occur in various organs throughout the body. Cardiotoxicity is one of the rare but serious irAEs that cannot be ignored for its extremely lethal. Through a review, Gan et al. and colleagues summarized the latest epidemiological evidence on the cardiovascular toxicity of programmed cell death protein-1 (PD-1)/programmed cell death ligand-1(PD-L1) inhibitors and the clinical manifestations, as well as the potential pathological mechanisms. Providing a novel perspective for monitoring early toxicity and establishing appropriate treatment for patients with ICI-related cardiotoxicity.

Another article with a focus contributed by Zhang et al. concentrated on Pancreatic injury (PI). Zhang et al. conducted a systematic review and meta-analysis to assess the incidence of PI in cancer patients who received ICIs in randomized controlled trials (RCTs) and found that patients treated with multiple ICIs had a higher chance of developing PI than those who received a single ICI. Moreover, although the incidence of ICIs-PI is not high, they are usually severe (≥ grade 3 events).

The occurrence of adverse effects has also been shown to correlate with the type of immunosuppression. Huang et al. provided convincing data from the real world to support this conclusion. They collected clinical information from 362 patients who developed different types of solid tumors and were treated with different ICIs and found that 29.24% of the patients discontinued immunotherapy due to irAEs, with pneumonitis being the main reason for discontinuation. The incidence of irAEs related to sintilimab and pneumonitis caused by pembrolizumab was higher. These data indicate the importance of having different monitoring priorities for different PD-1 inhibitors.

Another remarkable article is a case report provided by Zhao et al. The article reported on a 32-year-old patient who was diagnosed with stage III renal cell cancer (RCC) and received an immune checkpoint inhibitor (camrelizumab) in combination with tyrosine kinase inhibitor (axitinib) treatment for 1 year. The RCC diagnosed in this patient is a rare subtype of a renal tumor with Xp11.2 translocation/TFE3 gene fusions. After the adjuvant therapy of immune checkpoint inhibitor and tyrosine kinase inhibitor, the patient achieved a clinical complete response with no sign of recurrence or metastasis.

Also addressing renal tumors, Wang et al. mentioned that ICIs are only effective in some clear cell renal cell carcinoma (ccRCC) patients and can produce a wide range of immune-related adverse reactions. Unlike other neoplasms, the common biomarkers and degree of immunological infifiltration of ccRCC cannot predict the response of ccRCC to immunotherapy. Thus, Wang et al. integrated PBRM1 mutation data, transcriptome data, endogenous retrovirus data, and gene copy number data from 123 patients with advanced ccRCC who participated in prospective clinical trials of PD-1 inhibitors and established clinical prediction models. This model can accurately predict overall survival, progression-free survival, and response to immunotherapy of ccRCC.

Following are three large-scale studies targeting different cancer types. Firstly, Qian et al. reviewed seven eligible RCTs comparing ICI therapy alone or in combination versus other therapies in EGFR-TKI resistant NSCLC patients and found that ICI-based combination therapy had better PFS compared with those receiving conventional chemotherapy, indicating that this therapy could be offered to patients with EGFR-mutant NSCLC after progression following TKI treatment.

Secondly, Qi et al. focused on the efficacy and safety during the use of anti-PD1PDL1 monotherapy in progressive breast cancer. By reviewing a total of 586 patients with progressive breast cancer who received monotherapy with PD1PDL1 inhibitors in six studies, they concluded that anti–PD-1/PD-L1 monotherapy showed a manageable safety profile and had a promising and durable anti-tumor efficacy in metastatic breast cancer patients, and higher PD-L1 expression may be closely correlated to better clinical efficacy.

The third large-scale transcriptomic data Analysis conducted by Wang et al. investigated the role of lactate at the transcriptome level and its correlation with the clinical outcome of breast cancer (BC) and thyroid cancer (TC). They collected transcriptome data and clinical and somatic mutation data from 1,217 breast cancer patients and 568 thyroid cancer patients through The Cancer Genome Atlas (TCGA) and The Cancer Genome Atlas (GEO) and discovered that the lactate metabolism score was an independent prognostic factor and could serve as a reliable predictor of overall survival, clinical characteristics, and immune cell infifiltration, with the potential to be applied in immunotherapy or precise chemotherapy of BC and TC.

The incorporated studies of the Research Topic are very pertinent and well-fulfilled. They introduced the latest concepts and insights gained by experts in several cancer fields as they explore the occurrence and mechanisms of immune adverse reactions. With the efforts of these expert teams, the use of PD1/PDL1 inhibitors has been strongly promoted and the clinicians’ awareness of the immune adverse effects management has been increased. Hopefully, more new research and strategies which may guarantee both safety and efficacy of immunosuppressive drugs will emerge in the future, and more beneficial outcomes for patients may gain.

